# Early response predicts subsequent response to olanzapine long-acting injection in a randomized, double-blind clinical trial of treatment for schizophrenia

**DOI:** 10.1186/1471-244X-11-152

**Published:** 2011-09-23

**Authors:** Haya Ascher-Svanum, Fangyi Zhao, Holland C Detke, Allen W Nyhuis, Anthony H Lawson, Virginia L Stauffer, William Montgomery, Michael M Witte, David P McDonnell

**Affiliations:** 1 Global Health Outcomes, Eli Lilly and Company; Lilly Corporate Center, DC 4133; Indianapolis, IN, 46285 USA; 2 Psychosis Team, Eli Lilly and Company; Lilly Corporate Center, DC 6156; Indianapolis, IN 46285 USA; 3 Lilly Neuroscience, Lilly USA LLC; Indianapolis, IN, USA; 4 Eli Lilly Australia Pty Ltd; West Ryde, NSW, Australia; 5 Eli Lilly and Company; Cork ELCL, UK

## Abstract

**Background:**

In patients with schizophrenia, early non-response to oral antipsychotic therapy robustly predicts subsequent non-response to continued treatment with the same medication. This study assessed whether early response predicted later response when using a long-acting injection (LAI) antipsychotic.

**Methods:**

Data were taken from an 8-week, randomized, double-blind, placebo-controlled study of olanzapine LAI in acutely ill patients with schizophrenia (n = 233). Early response was defined as ≥30% improvement from baseline to Week 4 in Positive and Negative Syndrome Scale (PANSS_0-6_) Total score. Subsequent response was defined as ≥40% baseline-to-endpoint improvement in PANSS_0-6 _Total score. Sensitivity, specificity, positive predictive value (PPV), negative predictive value (NPV), and predictive accuracy were calculated. Clinical and functional outcomes were compared between Early Responders and Early Non-responders.

**Results:**

Early response/non-response to olanzapine LAI predicted later response/non-response with high sensitivity (85%), specificity (72%), PPV (78%), NPV (80%), and overall accuracy (79%). Compared to Early Non-responders, Early Responders had significantly greater improvement in PANSS_0-6 _Total scores at all time points and greater baseline-to-endpoint improvement in PANSS subscale scores, Quality of Life Scale scores, and Short Form-36 Health Survey scores (all p ≤ .01). Among Early Non-responders, 20% demonstrated response by Week 8. Patients who lacked early improvement (at Week 4) in Negative Symptoms and Disorganized Thoughts were more likely to continue being non-responders at Week 8.

**Conclusions:**

Among acutely ill patients with schizophrenia, early response predicted subsequent response to olanzapine LAI. Early Responders experienced significantly better clinical and functional outcomes than Early Non-responders. Findings are consistent with previous research on oral antipsychotics.

**Clinical Trials Registry:**

F1D-MC-HGJZ: Comparison of Intramuscular Olanzapine Depot With Placebo in the Treatment of Patients With Schizophrenia http://clinicaltrials.gov/ct2/show/NCT00088478?term=olanzapine+depot&rank=3

Registry identifier - NCT00088478

## Background

The time-course of response to antipsychotic therapy has been extensively evaluated, and the original belief that it took between 6 and 8 weeks to determine a therapy's effectiveness in patients with schizophrenia has largely been abandoned [1]. In many studies, most of the symptomatic improvement seen in response to atypical antipsychotic therapy occurs in the first 2 to 4 weeks, with effects seen in some patients in as early as 24 hours [1-3]. This finding has important implications for the medical management of acutely ill patients with schizophrenia who, individually, have an unpredictable response to any given antipsychotic. When symptom improvement is minimal or absent, adjustments in dose, delivery schedule, or drug therapy can be made sooner than once thought. This reduces unnecessary exposure to ineffective treatment while effective treatment is delayed and, in addition, may reduce the long-term clinical, functional, and economic harm associated with inadequate treatment [4].

Recently, there has been much interest in early response or non-response to treatment as a predictor of subsequent response to the same treatment. Failure to respond in the first 1 to 2 weeks of therapy has been shown to robustly predict later failure to respond to continued use of the same medication, a finding seen both in patients with chronic schizophrenia [5-8]. and in patients who are early in their course of illness [9]. Most of these analyses were retrospective, but a recent randomized, prospective study of early response produced similar results [7]. Although study designs have differed, and the analyses have involved different patient populations and utilized different definitions of and time points for assessing early and later response, the predictive power of early non-response resulting in later non-response has been a constant. Also constant has been the use of oral antipsychotic agents; the predictive strength of early response to depot (injectable) formulations has not yet been evaluated.

The trajectory of symptomatic improvement for patients who respond early to oral antipsychotic medication clearly differs from that of patients who do not show early response. The early responder groups have had higher baseline illness severity, more rapid symptom improvement, and consistently greater magnitude of improvement over time [6,7]. Investigators have identified characteristics at baseline that differentiate these distinct responder groups. For example, in analyses of patients with chronic schizophrenia who were treated with oral antipsychotics, early responders had, on average, shorter illness duration [6].; fewer acute exacerbations of schizophrenia in the previous 24 months [7].; higher likelihood of having schizoaffective disorder [6,7].; and higher Positive and Negative Syndrome Scale (PANSS) [10]. Total,[6,7]. Positive,[6,7]. and General Psychopathology [6]. scores than Early Non-responders. In patients with first-episode psychosis being treated with oral antipsychotics, Early Responders had shorter durations of illness and higher average weight at baseline than Early Non-responders [9]. Such differences may help clinicians optimize the initial choice of treatment for a given patient.

Whether patients who do or do not respond early to injectable antipsychotics differ in their functional outcomes or sense of health status also is not known. There is evidence based on analyses of response to oral antipsychotics to suggest that differences in functional outcome may exist. In a post hoc analysis of a 1-year, randomized, open-label study of oral antipsychotics, patients who demonstrated early response had greater improvement from baseline to Week 8 in measures of mental, physical, and functional health than those who did not respond early [4]. Similar degrees of functional improvement were also seen in a 12-week, prospective study of early response [7].

It is not known whether the predictive power of early response to oral antipsychotic therapy extends to long-acting injection (LAI) antipsychotics. Olanzapine LAI has been shown to have an early onset of action, with clinical improvement detectable as early as the first week of treatment. Although olanzapine LAI provides therapeutic drug levels following the first injection, drug levels continue to accumulate, reaching steady state olanzapine plasma concentrations approximately 3 months after initiation of therapy [10]. Given that olanzapine LAI takes longer to reach steady state than oral olanzapine, and given that it is dosed as frequently as every 2 weeks and as infrequently as every 4 weeks, it is not known what might constitute "early" response and whether early response would be predictive of subsequent response. In addition, it is unclear whether patients who do not initially respond to olanzapine LAI are likely to respond with further treatment. Because use of olanzapine LAI may have involved overcoming patient resistance to an injectable formulation, or may be viewed as a patient's "last resort," it is important not to discontinue therapy prematurely.

The primary objective of this analysis is to assess whether response to olanzapine LAI at Week 4 of treatment predicts response/non-response at Week 8 of treatment in acutely ill patients with schizophrenia. Secondary objectives include identifying baseline characteristics that might help differentiate Early Responders from non-responders, searching for differences in functional outcome between Early Responders and Early Non-responders, and identifying factors that predict later response in those patients who do not respond early.

## Methods

### Patient Population and Study Design

This was a post hoc analysis of data from an 8-week, randomized, double-blind trial comparing multiple dosing regimens of olanzapine LAI with placebo for treatment of acutely ill patients with schizophrenia [11]. Patients were male or female, aged 18 to 75 years, with a diagnosis of schizophrenia (DSM-IV or DSM-IV-TR), and a PANSS-derived Brief Psychiatric Rating Scale (BPRS, 0-6 scale) score ≥30, reflecting a moderate-to-high level of illness severity. Patients were excluded if they had experienced serious adverse events while taking oral olanzapine, currently had significant suicidal or homicidal tendencies, were currently pregnant or breast-feeding, or had a serious or unstable medical condition in the previous 30 days. The study was conducted from June 2004 to April 2005 at multiple study sites in the United States, Russia, and Croatia. Guidelines from the Declaration of Helsinki were followed, the study protocol was approved by local ethical review boards, and all patients or their authorized representatives signed written informed consent prior to participation in the trial (F1D-MC-HGJZ; NCT00088478; http://clinicaltrials.gov/ct2/show/NCT00088478?term=olanzapine+depot&rank=3).

After a 2- to 7-day screening and washout period, patients were randomly assigned (1:1:1:1) to receive olanzapine LAI in doses of 210 mg/2 weeks, 300 mg/2 weeks, 405 mg/4 weeks, or placebo. Patients entered as inpatients or outpatients, but were hospitalized during the wash-out period and for the first 2 weeks of treatment. If a patient had previously received depot antipsychotics, the interval since the last dose had to be 3 weeks or 1 injection interval, whichever was longer. Concomitant use of central nervous system medication was prohibited, except for limited use of select benzodiazepines/sedatives as needed for sleep.

### Assessments and Definitions

The primary outcome was mean baseline-to-endpoint change (last observation carried forward) in PANSS Total score after 8 weeks of treatment. The PANSS is a 30-item scale with each item scored between 1 (no symptoms) and 7 (severe symptoms), from which are derived a Total score, and Positive, Negative, and General Psychopathology subscores. The PANSS was administered at baseline, Day 3, Day 7, and weekly thereafter. Patients were assessed with the Short Form-36 Health Survey (SF-36) [12]. and the Quality of Life Scale (QLS) [13]. at baseline and Endpoint (Week 8). The SF-36 is a 36-item instrument for measuring health status and outcome from the patient's point of view. Scoring yields 8 health scores (vitality, physical functioning, bodily pain, general health perceptions, physical role functioning, emotional role functioning, social role functioning, and mental health) and 2 summary scores - the Mental composite score and the Physical composite score. The QLS is a 21-item scale that consists of a semi-structured interview administered by a trained clinician and provides information on symptoms and functioning during the preceding 4 weeks. Each item is scored from 0 to 7, with the low end of the scale reflecting more severe impairment. Scale items are grouped conceptually to form four subscales: Intrapsychic Foundations; Interpersonal Relations; Instrumental Role; and Common Objects and Activities.

"Early response" was defined as a ≥30% improvement over the baseline PANSS_0-6 _Total score at Week 4, and "later response" was defined as a ≥40% improvement over the baseline PANSS_0-6 _Total score at Week 8. Using these thresholds, patients were identified as "Early Responders" or "Early Non-responders" and "Late Responders" or "Late Non-responders." As suggested by Leucht et al. [14]. we rescaled the PANSS_1-7 _so that the range of scores was 0 to 6 rather than 1 to 7, such that the minimum score (ie, no symptoms) for the PANSS_0-6 _was 0 rather than 30. Rescaling of the PANSS_1-7 _prevented an underestimate of percentage improvement, since a 100% reduction in PANSS score is not possible using the other scaling convention [15].

### Statistical Analysis

This analysis included the 233 patients who received olanzapine LAI and who had a PANSS Total score at Week 4 and at least 1 PANSS Total score after Week 4. Conditional probabilities of early response (Week 4) predicting later response (Week 8) were calculated and included sensitivity, specificity, negative predictive value (NPV), positive predictive value (PPV), and total accuracy. To assess for differences between Early Responder and Early Non-responder groups, baseline characteristics for each group were compared using Fisher's exact test for categorical variables and t-test for continuous variables.

Least square (LS) mean change from baseline in PANSS Total score was compared between responder groups at each time point using a mixed model repeated measures method with baseline PANSS Total score, investigator, responder status, week, and responder status-by-week interaction terms.

Baseline-to-endpoint change in the Davis factor subscales of the PANSS were compared between Early Responder and Early Non-responder groups using an analysis of covariance model that included baseline score, investigator, and responder status. In 2001, Davis et al. [16]. used factor analysis to assign the 30-item PANSS to 5 subscales. The "Davis subscales" are weighted sums of specific PANSS items and include the following factors: Positive Symptoms (P1, P2, P3, P5, P6, P14, P23, P26, P29), Negative Symptoms (P8, P9, P10, P11, P13, P21, P30), Disorganized Thoughts (P2, P12, P14, P18, P19, P24, P25, P26, P27, P29), Impulsivity/Hostility (P4, P7, P22, P28), and Anxiety/Depression (P15, P16, P17, P18, P20). QLS measure subscores and SF-36 factor scores were compared between Early Responder and Early Non-responder groups using an analysis of covariance model that included baseline score of the measure being compared, investigator, and responder status.

For all analyses, results were considered significant at the p < .05 level and all statistical analyses were conducted using SAS (version 8.2, Cary, NC, USA).

## Results

The original study enrolled 404 patients, 306 of whom were treated with olanzapine LAI. Of the 306 patients, 233 (76%) met eligibility criteria for this analysis. Of these patients, 137 (58.8%) were identified as Early Responders and 96 (41.2%) were identified as Early Non-responders. The baseline characteristics of the Early Responder and Early Non-responder groups are shown in Table 1. There were no significant differences between responder groups on any demographic variable. The majority of patients were white, approximately one-third were female, and the average age was close to 40 years. Differences were found between groups regarding baseline psychopathology, with the Early Responder group appearing more ill at baseline. The mean PANSS Total score of Early Non-responders exceeded that of Early Non-responders by 5 points (102.5 vs. 97.5, p < .01). A significant difference was also observed for anxiety and depression between the Early Responder group compared with the Early Non-responder group (14.4 vs. 13.1, p=.005). The difference between scores for the PANSS Negative subscale also approached significance (24.8 for Early Responders, 23.3 for Early Non-responders; p=.05).

**Table 1:** Baseline Characteristics for Responder Groups^a ^among patients with chronic schizophrenia treated with olanzapine LAI

Baseline Characteristics	Early Responders (n = 137)	Early Non-responders (n = 96)	p-value
Age, years (mean [SD])	40.8 (11.6)	38.8 (11.1)	.18
Female gender, (n [%])	35 (25.6)	30 (31.3)	.34
Race (n [%])			.75
White	81 (59.1)	55 (57.3)	
African American	47 (34.3)	33 (34.4)	
Hispanic	8 (5.8)	5 (5.2)	
Other	1 (0.7)	3 (3.1)	
Age at first schizophrenia episode, years (mean [SD])	23.7 (7.9)	22.1 (8.7)	.15
PANSS scores (mean[SD])			
Total	102.5 (16.0)	97.5 (12.3)	.01
Positive	27.4 (5.5)	26.4 (4.5)	.14
Negative	24.8 (5.7)	23.3 (5.5)	.05
Disorganized Thoughts	25.0 (5.5)	24.1 (4.2)	.21
Impulsivity/Hostility	11.0 (3.5)	10.6 (3.4)	.36
Anxiety/Depression	14.4 (3.6)	13.1 (3.4)	.005
CGI-S mean [SD])	4.8 (0.8)	4.7 (0.7)	.20
Weight, kg (mean [SD])	85.6 (22.2)	84.1 (19.2)	.58

Abbreviations: CGI-S = Clinical Global Impression-Severity; PANSS = Positive and Negative Syndrome Scale; SD = standard deviation.

^a ^Early response = ≥30% reduction from baseline in PANSS Total score at Week 4; later response = ≥40% reduction in PANSS Total score at Week 8.

**Figure 1:**
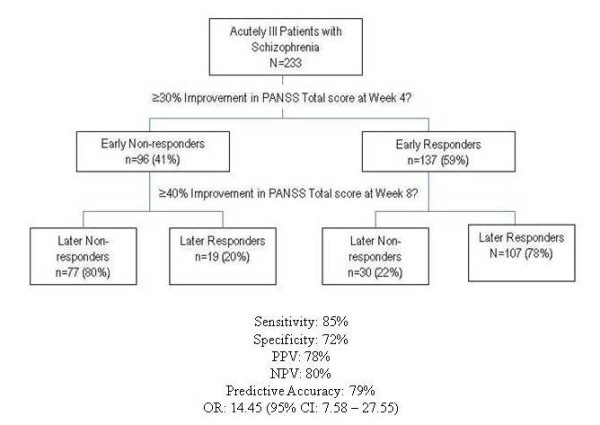
**Model and conditional probabilities for early response predicting later response for patients with chronic schizophrenia**. Abbreviations: CI = confidence interval; NPV = negative predictive value; OR = odds ratio; PPV = positive predictive value. Definitions: early response = ≥30% reduction from baseline in PANSS Total score at Week 4; later response = ≥40% reduction in PANSS Total score at Week 8.

Response at Week 4 predicted response at Week 8 with a good degree of accuracy. Sensitivity was 84.9%, specificity was 72.0%, PPV was 78.1%, NPV was 80.2%, and total accuracy was 79.0% (Figure 1). Rates of study discontinuation for any reason were higher for Early Non-responders than for Early Responders (25.0% versus 17.5%, p=.007).

**Figure 2:**
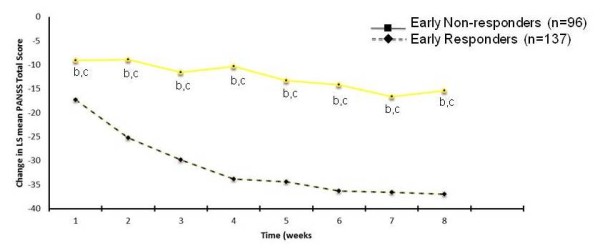
**Least square mean change from baseline in PANSS Total score for Responder Groups**. Abbreviations: PANSS = Positive and Negative Syndrome Scale Score. a Early response was defined as a ≥30% improvement from baseline in PANSS Total score at Week 4. b Statistically significant within-group difference, p < .001. c Statistically significant between-group difference, p < .001.

LS mean change from baseline in PANSS Total score for patients identified as Early Responders and Early Non-responders is shown in Figure 2. Early responders had significantly greater change in PANSS Total score at every time point (p < .001), and by Week 8, they had over twice the reduction in LS mean PANSS Total score than Early Non-responders had (37.0 points versus 15.4 points).

**Figure 3:**
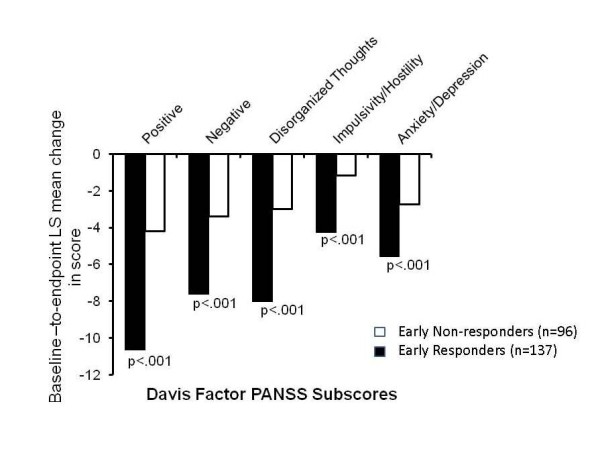
**Least square baseline-to-endpoint mean change in Davis Factor PANSS subscale scores for Responder Groups**. Abbreviations: LS = least square; PANSS = Positive and Negative Syndrome Scale Score. a Early response was defined as a ≥30% improvement from baseline in PANSS Total score at Week 4.

Baseline-to-endpoint LS mean change in Davis Factor PANSS subscale scores for patients identified as Early Responders and Early Non-responders is depicted in Figure 3. For all 5 PANSS subscale scores - Positive, Negative, Disorganized Thoughts, Impulsivity/Hostility, and Anxiety/Depression - Early Responders had greater baseline-to-endpoint improvement than Early Non-responders at a p < .001 level of significance. For each subscale, change in PANSS subscale for the Early Responder group was at least double that of the Early Non-responder group.

With regard to the patients' sense of health status, Early Responders had significantly more improvement in the following LS mean SF-36 subscale scores: Mental Component Summary (p=.01), Mental Health (p=.004), and Social Functioning (p=.002). On the QLS, Early Responders had significantly greater improvement than Early Non-responders for the Total score (p < .001) and all subscales (Common Objects and Activities, p=.01; Intrapsychic Foundations, p < .001; Interpersonal Relations, p < .001; and Instrumental Role, p=.004).

**Figure 4:**
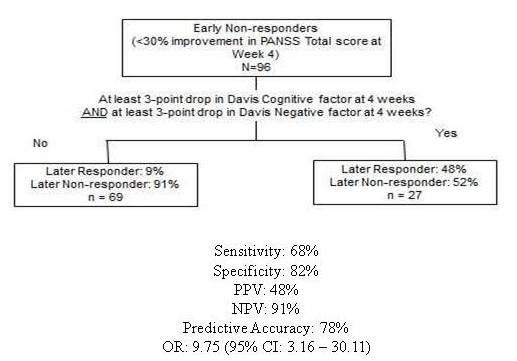
**Conditional probabilities for predicting later response in Early Non-responder patients (N = 96)**. Abbreviations: CI = confidence interval; NPV = negative predictive value; OR = odds ratio; PPV = positive predictive value. Definitions: later response = week 8; early response = week 4.

Of the 96 patients who did not meet criteria for response at Week 4, 19 patients (20%) met criteria for subsequent response. In the regression model, lack of improvement in the Negative Symptom and the Disorganized Thoughts factors predicted non-response at endpoint for patients who had not responded by Week 4. Specifically, lack of at least a 3-point drop from baseline in both of these factor scores at Week 4 predicted continued non-response with a 48% PPV, 91% NPV, 68% sensitivity, and 82% specificity (Figure 4). The Negative Symptom factor includes measures of blunted affect, emotional withdrawal, poor rapport, passive/apathetic social withdrawal, flow of conversation, motor retardation, and active social avoidance. The Disorganized Thoughts factor includes assessment of conceptual disorganization, difficulty in abstract thinking, stereotyped thinking, tension, mannerisms and posturing, disorientation, poor attention, lack of judgment and insight, disturbance of volition, and preoccupation.

## Discussion

In this post hoc analysis of data from a randomized, double-blind, clinical trial of olanzapine LAI for treatment of acute exacerbation of schizophrenia, early response (Week 4) was found to predict subsequent response (Week 8) with high degrees of sensitivity, specificity, PPV, and NPV, and an overall accuracy level of 79%. The group of patients who met criteria for early response had a trajectory of symptom recovery that was distinct from that of the group who did not meet response criteria. Compared to Early Non-responders, Early Responders had a higher level of symptom severity at baseline as measured by PANSS Total score, and had a greater degree of improvement over baseline at every subsequent time point. By Week 8, improvement by Early Responders was more than double that of Early Non-responders (-37 points vs. -15 points).

The total accuracy of predicting later response to olanzapine LAI based on early response exceeded that seen in studies of oral antipsychotics, including analyses of patients with chronic schizophrenia,[7]. of patients with first-episode psychosis,[9]. of a prospective study of patients with chronic schizophrenia,[7]. and of 5 pooled studies of patients with chronic schizophrenia [6]. (79% for olanzapine LAI versus 72%, 70%, and 74%, respectively). Much of the increase in total accuracy was due to stronger performance for sensitivity (85% for olanzapine LAI versus 70%, 77%, and 60%, respectively) and PPV (78% for olanzapine LAI versus 63%, 42%, and 54%, respectively). Stronger predictive characteristics in this analysis may have been due to use of a later time point to assess early response; Week 4 for this analysis versus Week 2 in the oral olanzapine studies. Predictions of later response have been shown to improve as the time point for early assessment is delayed,[17]. and a balance must be struck between improved predictive accuracy and expedient recognition of a need to adjust treatment. In this instance, the later time point was chosen because for many of the patients in this study, 4 weeks represented the interval between injections, and was therefore the earliest point at which treatment could be re-evaluated. Another possible explanation for why the predictive accuracy seen in this study of olanzapine LAI was better than that seen in studies of oral antipsychotics is that with depot formulations, adherence is guaranteed at least through the injection interval. Adherence to oral antipsychotics is not assured. Study populations containing a mix of patients with varying degrees of adherence may have lowered the predictive accuracy of early response/non-response in those studies.

A comparison of rates of study discontinuation between Early Responders and Early Non-responders was included in this analysis because this outcome is associated with increased rates of relapse and psychiatric hospitalization, decreased functional outcome and quality of life, and increased treatment costs [15,18-22]. Moreover, in a response trajectory analysis, treatment response was closely aligned with discontinuation rates [23]. While the relationship between short-term and longer-term persistence with therapy and the clinical significance of short-term persistence are not known, results presented here suggest that differences in efficacy noted as early as Week 2 may be an important determinant of short-term persistence with treatment.

Early Responders to olanzapine LAI differed significantly from Early Non-responders at baseline by having higher PANSS Total scores. In prior studies of oral antipsychotics for treatment of patients with chronic schizophrenia, Early Responders also had higher baseline PANSS Total scores than Early Non-responders,[6,7]. though this difference was not found for patients with first-episode psychosis [9]. Other baseline discriminators between Early Responders and Early Non-responders have been identified, including differences in PANSS Positive [6,7]. and General Psychopathology [6]. subscores. However, a difference in the PANSS Negative subscore, as was seen in this study, had not been noted previously. It appears that individuals with higher levels of baseline psychopathology may have higher likelihoods of responding to treatment, whether given orally or in a depot formulation. Determining a cut-off for the baseline PANSS score that maximized the predictive value of this assessment tool could help clinicians optimize treatment.

Early improvement in symptoms of schizophrenia in response to olanzapine LAI not only predicted later response in a total measure of symptoms of schizophrenia, but also predicted baseline-to-endpoint improvement across multiple symptom domains and other measures of quality of life, mental health, and social functioning. This study highlights potential benefits to patients, clinicians, families, and payers of early evaluation and identification of patients responding poorly to treatment. These findings echo those of Ascher-Svanum et al.,[4]. who found that in a post hoc analysis of data from a 1-year, randomized, open-label study of antipsychotic therapy for treatment of schizophrenia, patients who demonstrated response at Week 4 had greater improvement from baseline to Week 8 in measures of mental health, emotional role social functioning, physical functioning, vitality, and a composite mental health score than those who did not respond at Week 4. Likewise, in a randomized, double-blind, flexible-dosed, prospective 12-week study of people with schizophrenia treated with oral risperidone therapy, patients with ≥20% improvement on the PANSS Total score by Week 4 of treatment achieved significantly greater baseline-to-Week 12 improvement across all functional subdomains of the Schizophrenia Objective Functioning Instrument, a validated functional measure including subdomains of living situation, instrumental activity, productive activity, and social functioning [7].

Of the patients who did not meet criteria for response at Week 4, 1 in 4 went on to meet the criteria for response at Week 8. A regression model to identify characteristics that distinguish Early Non-responders who do and do not respond at a subsequent time point found that lack of early improvement in 2 PANSS subscale scores - Negative Symptoms and Disorganized Thoughts - predicted continued non-response at endpoint. Specifically, lack of at least a 3-point drop in the Negative subscale score and lack of at least a 3-point drop in the Disorganized Thoughts subscale score at 4 weeks predicted continued non-response. Using this secondary predictive model as a confirming diagnostic test, lack of subsequent response could be predicted with near certainty (NPV = 91%). This secondary prediction model could potentially help in situations where clinicians need reassurance before proceeding with a change in treatment strategy.

This analysis had several limitations that should be noted. First, it focused on a sample that consisted predominantly of inpatients who were acutely, markedly ill at baseline and had a history of multiple relapses. Results cannot be extrapolated to patients with less severe symptomology or who are earlier in their course of illness. This analysis used data from a trial that was 8 weeks in length. Further study is needed to assess this phenomenon over a longer treatment period. Also, the 30-item PANSS used to assess early response/non-response is a lengthy measure that is not used in usual clinical practice, thus potentially limiting the clinical applicability of our findings. However, we have recently identified 6 PANSS items that can be used to reliably assess response/non-response to oral antipsychotics with only a slight loss in predictive accuracy [24]. Whether an abbreviated assessment such as this can be used to predict longer term response based on early response to olanzapine LAI will need to be determined. Finally, the early and substantial improvement seen in patients with the more severe pathology at baseline may be simple regression toward the mean, but this is a limitation of all analyses of this type.

## Conclusions

In the course of treatment of acutely ill patients with schizophrenia, early response to olanzapine LAI was found to predict later response with a high degree of accuracy, similar to that seen with oral antipsychotics. Although patient demographics were not helpful in distinguishing Early Responders from Early Non-responders, greater baseline symptom severity, particularly in the depressive and negative symptom domains, did help identify those patients more likely to respond early. Future analyses may want to further examine the predictive value of these factors, as they have utility in identifying response trajectories, as well [25]. Nearly one quarter of patients who did not meet criteria for response at Week 4 demonstrated response by Week 8. Patients who lacked early improvement (at Week 4) in Negative Symptoms and Disorganized Thoughts were more likely to continue being non-responders at Week 8. Compared to Early Non-responders, the Early Responders were not only more likely to respond to continued treatment with olanzapine LAI, but were also more likely to experience greater improvements in functional outcomes and persist longer on therapy. An early response to antipsychotic medication may serve as an early clinical marker that clinicians can use when balancing the benefit and risk of treatment with olanzapine LAI for acutely ill patients with schizophrenia.

## Competing interests

Drs. Ascher-Svanum, Zhao, Detke, Stauffer, Witte, and McDonnell, and Mr. Nyhuis, Lawson, and Montgomery are full-time employees of Eli Lilly and/or any of its subsidiaries, and minor stockholders of Eli Lilly and Company.

## Authors' contributions

All authors contributed to the development and content of the manuscript, and approved the final version, and qualify for authorship under the International Committee of Medial Journal Editor's Uniform Requirements for Manuscripts Submitted to Biomedical Journals (NEJM 1997(336):309-315 guidelines).

## Pre-publication history

The pre-publication history for this paper can be accessed here:

http://www.biomedcentral.com/1471-244X/11/152/prepub
